# FedPSO: Federated Learning Using Particle Swarm Optimization to Reduce Communication Costs

**DOI:** 10.3390/s21020600

**Published:** 2021-01-16

**Authors:** Sunghwan Park, Yeryoung Suh, Jaewoo Lee

**Affiliations:** 1 The Department of Security Convergence Science, Chung-Ang University, Seoul 06974, Korea; tjdghks994@gmail.com (S.P.); ye0s3a@gmail.com (Y.S.); 2 The Department of Industrial Security, Chung-Ang University, Seoul 06974, Korea

**Keywords:** particle swarm optimization, federated learning, aggregation, convolutional neural network (CNN)

## Abstract

Federated learning is a learning method that collects only learned models on a server to ensure data privacy. This method does not collect data on the server but instead proceeds with data directly from distributed clients. Because federated learning clients often have limited communication bandwidth, communication between servers and clients should be optimized to improve performance. Federated learning clients often use Wi-Fi and have to communicate in unstable network environments. However, as existing federated learning aggregation algorithms transmit and receive a large amount of weights, accuracy is significantly reduced in unstable network environments. In this study, we propose the algorithm using particle swarm optimization algorithm instead of FedAvg, which updates the global model by collecting weights of learned models that were mainly used in federated learning. The algorithm is named as federated particle swarm optimization (FedPSO), and we increase its robustness in unstable network environments by transmitting score values rather than large weights. Thus, we propose a FedPSO, a global model update algorithm with improved network communication performance, by changing the form of the data that clients transmit to servers. This study showed that applying FedPSO significantly reduced the amount of data used in network communication and improved the accuracy of the global model by an average of 9.47%. Moreover, it showed an improvement in loss of accuracy by approximately 4% in experiments on an unstable network.

## 1. Introduction

Recently, the use of mobile devices such as smartphones and tablets has been increasing. Various forms of data are being generated and accumulated on mobile devices, including data generated by users and sensors such as cameras, microphones, and the global positioning system. The accumulated data on mobile devices are beneficial for deep learning, which demonstrates good performance when there is a significant amount of data.

Data from mobile devices can be used for machine learning (ML) in various ways [1]. Google’s Gboard, for example, uses ML to learn words that users frequently type and recommends the next words to be typed [2]. However, there are four points to consider in using mobile device data for ML.

- Mass data collection cost: network communication and storage costs for collecting and managing large amounts of original data on the server are high.
- Unstable networks: mobile devices cannot use wired networks and are mostly connected via Wi-Fi, making it difficult to establish a stable network environment.
- Low computation capability: the processors in mobile devices do not have sufficient computing capabilities for ML.
- Security threat: collecting or storing private data increases the likelihood of data breaches.

Therefore, to implement a successful ML model, especially an artificial neural network (ANN (ANN: Artificial Neural Network, a statistical learning algorithm inspired by biological neural networks among ML methods. Examples are supervised learning and unsupervised learning.)), using mobile device data, it is necessary to reduce the size of the collected data, strengthen the security of the collected data, improve the robustness to an unstable network environment, and reduce the number of training parameters (weight (Weight: The weight used in this paper refers to a parameter that updates the input data in the hidden layer of the ANN. In short, it is a value that determines the amount of influence input data has on output data. The weight is updated through the ANN’s back-propagation process as training progresses.) of neural network). Research on federated learning has been steadily progressing to solve these problems and be able to use the vast amount of data on mobile devices [3,4]. Federated learning is an ML model for training on distributed data. This model ensures privacy by not sending data from personal devices to a central server. In addition, federated learning reduces communication costs by transmitting only the learned models without transmitting large amounts of source data to the server.

In conventional ANN models, calculation time accounts for much more than communication time, so various algorithms are used to reduce the calculation time, such as using graphics processing unit (GPU) accelerators and connecting many GPUs. However, in federated learning, communication takes more time than calculation. Thus, network communication time should be reduced to improve the efficiency of federated learning. Because of unstable network environment problems, federated learning requires environmental conditions such as Wi-Fi connections and connected chargers [4]. Therefore, to reduce the communication cost of federated learning, it is necessary to improve network transmission speed and solve problems with unstable network environments.

Most of the models using federated learning use the global model federated averaging (FedAvg) [5]. The current study aims to increase the model update speed by applying Particle Swarm Optimization (PSO), which is an algorithm that obtains an optimal solution in a distributed environment [6,7]. PSO requires many repetitions because it obtains the optimal solution through a stochastic approach, which is in line with learning through many repetitions of ML. The PSO is well suited to dynamic and heterogeneous environments such as federated learning. Thus, we propose a new ANN model by applying the PSO to federated learning.

1. To the best of our knowledge, this paper is the first paper focused on reducing network communication costs by applying PSO in the communication process of federated learning. We propose a new model, federated PSO (FedPSO), that collects scores such as accuracy and loss rather than weights for global model updates.
2. We evaluate FedPSO for network communication cost and accuracy. In experimental results, the network communication cost of FedPSO was less than that of existing models, and FedPSO had an average accuracy improvement of 9.47%.
3. We evaluated FedPSO in an unstable network environment. In experimental results, the proposed FedPSO showed a 4% improvement in loss of accuracy over existing algorithms.

The rest of the paper is structured as follows. Section 2 reviews previous studies that use federated learning and PSO. Section 3 describes the process of transmitting the model learned from the client to the server through the proposed algorithm. The evaluation of our proposed technique is presented in Section 4, before finally concluding the paper in Section 5.

## 2. Background and Related Work

### 2.1. Federated Learning

Federated learning is a learning method proposed by Konecný et al. [3,4] for distributed datasets. It trains a model using datasets distributed across various devices while preventing data leakage. Federated learning is advantageous in that it improves privacy and reduces communication costs. Through federated learning, ANN models can learn without breaches of data or personal information. In addition, transferring all the data from numerous devices to a central server increases network traffic and storage costs. Federated learning significantly reduces communication costs by exchanging only the weights obtained from training the models.

**Figure 1:**
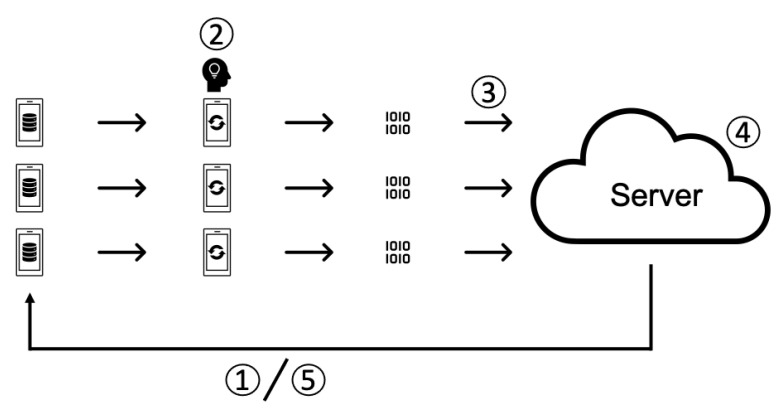
Federated Learning Protocol.

Figure 1 outlines the federated learning process.

1. The server sends the learning model to each client.
2. The received models are trained on client data.
3. Each client sends its trained model to the server.
4. The server processes the collected models and aggregates them into a single updated model.
5. The server sends updated models to each client, and steps 1 to 5 are repeated.

Many federated learning studies use algorithms such as federated stochastic gradient descent (SGD: Stochastic Gradient Descent; iterative method for optimizing an objective function.) (FedSGD) and federated averaging (FedAvg) to implement the fourth step in Figure 1. The algorithm was proposed by McMahan [5] and is used in several federated learning studies to update models collected on servers. Both algorithms obtain the parameters of the global model by obtaining the average values of the parameters collected from each client. FedSGD does not update the weights on the client but instead obtains the weights at the server. In this algorithm, the gradient is sent to the server to calculate the average, and the global weights are updated to create a global model. FedAvg is an algorithm that uses a combination of FedSGD and mini-batches to update models directly on the client, and the server averages the weights to create a new global model.

Federated learning assumes a distributed mobile device environment. Mobile devices have the disadvantage of having to learn in a wireless network environment, rather than a stable wired network connection. If the network is unstable, the client involved in learning may lose its connection or the client may not be able to send the complete dataset when sending the trained model.

### 2.2. Particle Swarm Optimization

PSO, the most well-known metaheuristic global optimization algorithm, was developed by Kennedy and Eberhart in 1995 [6,7]. The algorithm optimizes a number of variables at once with algorithms inspired by bird and fish swarms in nature. The PSO algorithm has advantages in memory requirements and speed that result from its easy implementation, scalability, robustness, quick convergence, and simple mathematical operations. The algorithm uses a probabilistic approach that requires a large number of iterations for optimization.

PSO components can be divided into the swarm and particles. A swarm consists of a set of particles. Each particle represents a possible solution to the problem. Each particle has a position and speed *V* for the next step. To find the global optimal value, particles communicate with each other step by step and share their own pbest (particle best) variable. Each particle sets the gbest (global best) variable to the optimal value of the shared pbest values: $gbest=\max_{i}\left(pbest\right)$. Each particle calculates the inertia ($V^{t-1}$, the speed of previous step), pbest, and gbest values using Equation (1) below to obtain the speed to move on to the next step.

(1) $$\begin{array}{c} V_{i}^{t}=\alpha \cdot V_{i}^{t-1}+c_{1}\cdot rand_{1}\cdot \left(pbest-V_{i}^{t-1}\right)+c_{2}\cdot rand_{2}\cdot \left(gbest-V_{i}^{t-1}\right) \end{array}$$

In Equation (1), α is a constant representing the inertia weight, c1 is the acceleration constant for the pbest, and c2 is the acceleration constant for gbest. The values of $rand_{1}$ and $rand_{2}$ are any random value between 0 and 1.

### 2.3. Related Work

There are many studies on communication between clients to improve federated learning performance. Federated learning has many problems arising from the unstable network environment of mobile devices, such as frequent node crashes, frequently shifting node groups, high central server overhead, and increased latency as the number of nodes increases. In addition, multi-layer models have been used to improve learning accuracy, but as the layers deepen, the number of weights for the nodes increases. Data size is a limitation for federated learning because it increases the size of the network transmission between the server and the client.

Recently, various studies have addressed this problem. To improve the network performance of federated learning, research has been conducted on low rank and random mask [4] and temporal weights [8]. However, these studies may reduce accuracy in unstable network environments.

The traditional federated learning model also has security threats. Federated learning models often send all the model weights to the server. Zhu et al. [9] showed that sending all the weights is potentially dangerous because confidential data can be extracted from reverse computation of the model weights transmitted over the network. Therefore, we focused on minimizing the collection of weights on the server.

Meanwhile, studies on the use of PSO algorithms in distributed environments are steadily progressing. Particles obtain the global optimal value at the same time. Studies have examined dynamic multi-swarm PSO (DMS-PSO) for preventing falling into local minima [10,11,12], PSO for neighbor selection in peer-to-peer (P2P) network environments [13], and gossip-based PSO for maintaining flexible P2P networks [14]. In addition, many studies enable PSO to be used in various distributed environments [15,16].

PSO has been applied to ML as well as research for performance improvement, such as a PSO convolutional neural network (PSO-CNN), which uses a PSO to classify images [17,18], linearly decreasing weight PSO (LDW-PSO) for convolutional neural network (CNN) hyperparameter optimization [19], and PSO and CNNs for lung nodule analysis [20]. Self-adapted particle swarm estimation of distribution algorithms (sa-PSEDA) apply PSO for optimization-driven prediction (ODP), a new classification method for automatic medical diagnosis and prognosis prediction [21]. Another paper used PSO enhanced with ANNs to solve complex problems in civil engineering [22]. A study used a deep learning neural network with PSO for gully erosion susceptibility [23]. In addition, a study applied PSO to improve the learning performance of federated learning clients by finding optimized hyperparameters [24]. As such, PSO has been steadily applied to various methods such as updating ML model weights or tuning hyperparameters.

Most of the previous papers focus on communication between clients and global optimization to improve the performance of federated learning. However, there has never been a study to maintain robustness to data transmission failures in the unstable network environment of federated learning. In addition, there have been attempts to apply PSO in various ways to federated learning, but PSO have never been used to improve the performance of global models through network communication performance improvement. We focus on improving the performance of federated learning by changing the form of data used in communication between servers and clients in a global model update method based on PSO.

## 3. FedPSO: Federated Particle Swarm Optimizing

**Figure 2:**
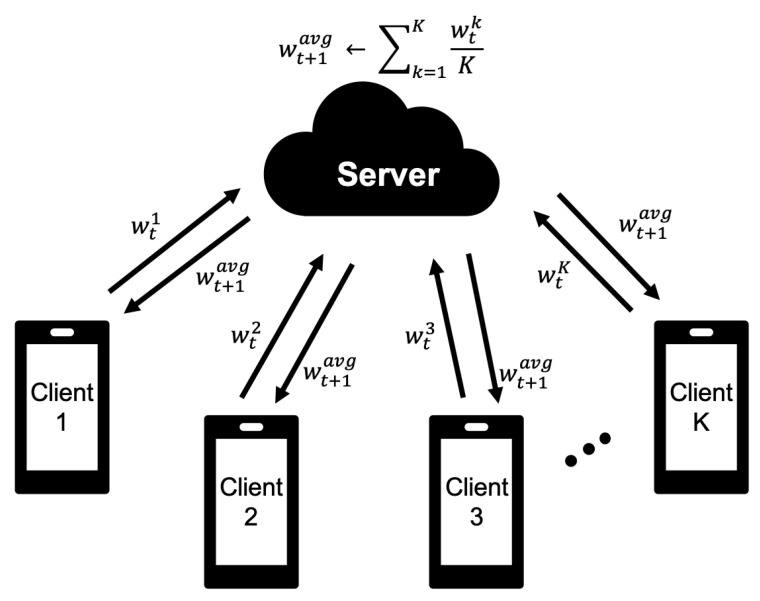
The weighted aggregation process of Federated Learning (such as FedAvg) obtains the average of the $w_{t}$ value received from the client of K from the server and sends the updated $w_{t}+1$ back to the client.

A general approach to improving the accuracy of ANN models is to deepen the layers of the model. This is called a deep neural network. As the layers become deeper, the number of weight parameters that require training increases. In the universal federated learning (as shown in Figure 2), when the model trained on the client is sent to the server, the network communication cost increases considerably. Therefore, we propose the FedPSO algorithm, which sends the best score (such as accuracy or loss) to the server by utilizing PSO characteristics to transmit the trained model, regardless of size.

Before explaining the proposed FedPSO, we will analyze the algorithm used in the previous work on federated learning (such as FedAvg [5]). The process of Algorithm 1 used in federated learning is as follows. The client participating in the round is selected through Line 4. The process of receiving the weight values learned from the client is accomplished through Lines 5 and 6. When the weight collection is completed, the average of the weights collected through Line 7 is calculated, and then the global weights are calculated. The client receives the global weights from the server and learns the data through Lines 8–10.
**Algorithm 1**FederatedAveraging (FedAvg) algorithm (simplified from [5]); K = number of clients; E = client total epochs; Select client by the *C* ratio.1:**function**ServerExecutes2:    initialize $w_{0}$3:    **for** each round t=1,2,… **do**4:        $S_{t}\leftarrow$ (random set of max(C·K,1) clients)5:        **for** each client $k\in S_{t}$ **in parallel do**6:            $w_{t+1}^{k}\leftarrow$ **ClientUpdate**(*k*, $w_{t}$)7:        $w_{t+1}\leftarrow$ (averaging of the collected weights $w_{t+1}^{k}$ of $S_{t}$ clients)                  8:**function**Client Update(*k*, *w*)9:     Perform learning process on client *k* with weight *w* until the client reaches *E* epoch10:   w← updated weight after learning11:   **return** *w* to server

**Figure 3:**
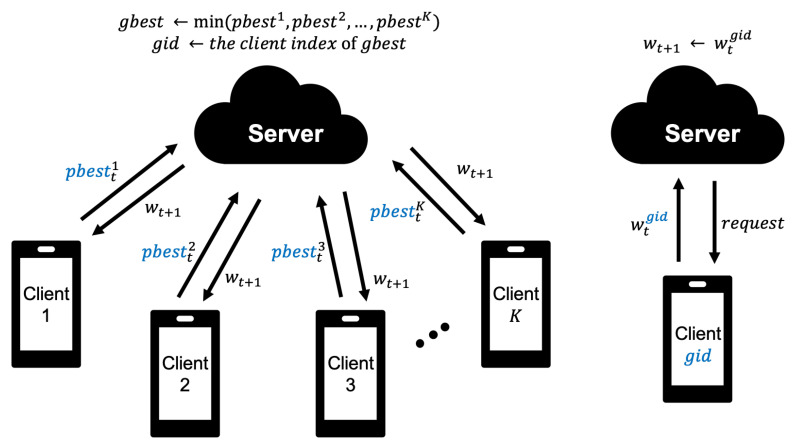
The weight update process of FedPSO; the server receives a client’s score and requests a learning model from the client who submits the optimal value to set it as a global model.

Next, the proposed model, FedPSO, receives the model weights only for the client that provided the best score so that the model weights do not need to be transmitted from all clients. The process is shown in Figure 3. The best score uses the lowest loss value derived after training on the client. This loss value is only 4 bytes. FedPSO identifies the best model through pbest and gbest variables and updates using the value of *V* for each weighted array element of the best model.

As the ANN weight values were updated in Equation (1), we can represent the weight update those for FedPSO as follows:

(2) $$\begin{array}{cc} V_{l}^{t}= & \alpha \cdot V_{l}^{t-1}+c_{1}\cdot rand_{1}\cdot \left(pbest-V_{l}^{t-1}\right)+c_{2}\cdot rand_{2}\cdot \left(gbest-V_{l}^{t-1}\right)\\ w_{i}^{t}= & w_{i}^{t-1}+V^{t} \end{array}$$

In Equation (2), V in ANN has a value for each layer of weight w. The current step weight $w^{t}$ is obtained by adding V to the previous step weight $w^{t-1}$. As in Equation (1), α is a constant representing the inertia weight, c1 is the acceleration constant for pbest, and c2 is the acceleration constant for gbest. The values of $rand_{1}$ and $rand_{2}$ are any random value between 0 and 1.

Based on the weight update equation (Equation (2)), we present the conceptual algorithm of FedPSO in Algorithm 2. The algorithm is extended based on Algorithm 1 applying PSO. Unlike conventional algorithms, Function ServerExecutes receives only pbest values, without receiving w from the client on Line 5. The task of finding the client with the minimum pbest value among those collected is executed through Lines 6–8. Function ClientUpdate proceeds the ANN applying the PSO. Lines 13–14 calculate Variable *V* used in the previous step, the optimal value of $w^{pbest}$ stored by the user, and the $w^{gbest}$ value received to the server. This process is carried out for each layer weight. Then, Variable *V* is added to the *w* from the previous round to calculate the *w* to be used in the current round through Line 15. After that, repeat the training through Lines 16–18 as many times as the client epoch *E*. Function GetBestModel is a function that requests the model from the client with the best score on the server (Lines 20–23).
**Algorithm 2**FedPSO1:**function**ServerExecutes2:    initialize $w_{0}$, pbest, gbest, gid3:    **for** each round t=1,2,… **do**4:        **for** each client k **in parallel do**5:           pbest ← **ClientUpdate**(*k*, $w_{t}^{gid}$)6:           **if** gbest>pbest **then**7:               gbest←pbest8:               gid←k         9:        $w_{t+1}\leftarrow$ **GetBestModel**(gid)         10:**function**ClientUpdate(*k*, $w_{t}^{gid}$)11:    initialize $V,w,w^{pbest},\alpha ,c_{1},c_{2}$12:    β← (split $\rho_{k}$ into batches of size *B*)13:    **for** each weight layer l=1,2,… **do**14:        $V_{l}\leftarrow \alpha \cdot V_{l}+c_{1}\cdot rand\cdot \left(w^{pbest}-V_{l}\right)+c_{2}\cdot rand\cdot \left(w_{t}^{gbest}-V_{l}\right)$15:    w←w+V16:    **for** each client epoch *i* from 1 to *E* **do**17:        **for** batch b∈B **do**18:           w←w−η∇l(w;b)             19:    **return** pbest to server         20:**function**GetBestModel(gid)21:    request to **Client**(gid)22:    receive *w* from **Client**23:    **return** *w* to server

## 4. Experiments

To evaluate the effectiveness of FedPSO, we conducted experiments to determine the accuracy and convergence speed and experiments in an unstable network environment. In the first experiment, we wanted to determine whether the model had sufficient accuracy and convergence speed, given its smaller amount of network communication than FedAvg. We used the Canadian Institute for Advanced Research (CIFAR-10) and Modified National Institute of Standards and Technology (MNIST) datasets for the accuracy benchmarks of the two algorithms and reviewed the cost of data communication between clients and servers. In the second experiment, we investigated the accuracy of FedPSO and FedAvg under various network environments.

### 4.1. Experimental Setup

We conducted the experiments on a server (desktop computer) with an AMD Ryzen 3950x CPU, two NVIDIA GeForce RTX 2070 Super GPUs with 8 GB DRAM each, and 64 GB memory. Our experimental code was written using TensorFlow version 2.3.0 and Keras version 2.4.3. The code is available in the FedPSO GitHub (FedPSO GitHub; https://github.com/tjdghks994/FedPSO).

The study was proposed to improve the network communication performance of federated learning. Thus, we updated the weights of the distributed model using the PSO and changed the form of the data sent by the client to the server. The CNN model produced high accuracy but was not used because it was complicated. Therefore, we conducted experiments using a two-layer CNN model (the first with 32 channels, the second with 64, each followed by 2 × 2 max pooling), the same as FedAvg [5]. The layers of the corresponding model are shown in Table 1.

The experiment was conducted using the CIFAR-10 and MNIST dataset. CIFAR-10 is a dataset frequently used for image classification. It consists of 32 × 32-pixel images from 10 classes such as airplane, automobile, and cat, and it has 50,000 training images and 10,000 test images. MNIST is another computer vision dataset used for image classification and verification. It consists of handwritten 28 × 28-pixel images of numbers, and it has 60,000 training images and 10,000 test images. Both datasets were shuffled, assigned to particle numbers, and distributed to each particle to proceed with training.

The separate tuning process to improve accuracy during the training process was not used except for the dropout layer. Both FedPSO and FedAvg used SGD methods for client training, and the learning rate value was 0.0025. The hyperparameter value used in the paper is also shown in Table 2.

### 4.2. Experimental Result for Accuracy

**Figure 4:**
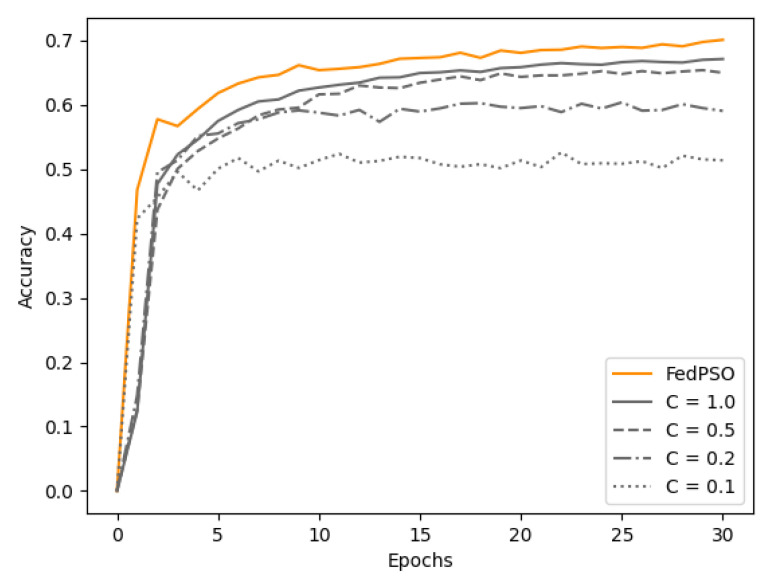
Accuracy comparison of several algorithm.

**Figure 5:**
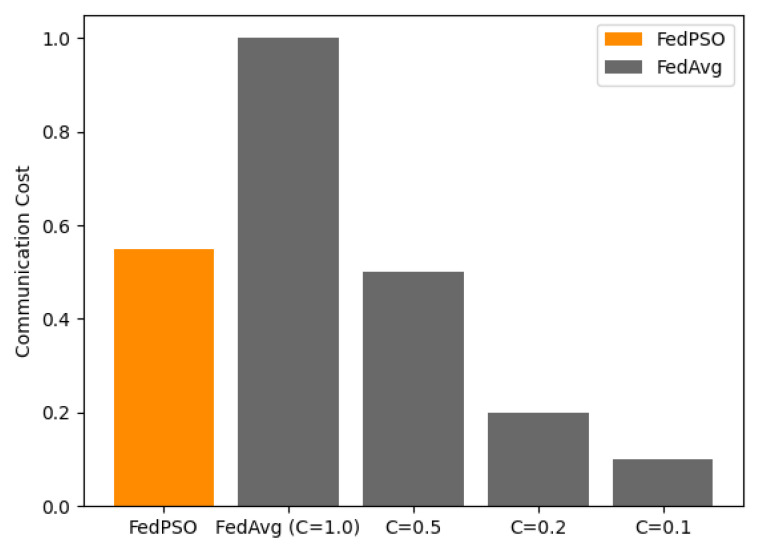
Communication Cost comparison of several algorithm.

The accuracy experimental results with the CIFAR-10 dataset are presented in Figure 4 and Table 3. All of these graphs were based on test accuracy. FedPSO produced a higher accuracy (70.12%) than FedAvg in all cases at 30 epochs, and it was more accurate from an early epoch. The highest accuracy of FedAvg was 67.14% at C=1.0. *C* is a constant between 0 and 1 that restricts the number of clients to be used for training in FedAvg. In each communication round, the experiment was conducted by selecting a client as high as *C* from all the clients. The higher the value of *C* in Figure 4 and Figure 5, the higher the accuracy, but the amount of data transmitted between the server and client increases accordingly. At C=0.5, which has similar data transfer costs, the difference in accuracy is greater (65.00% for FedPSO).

**Figure 6:**
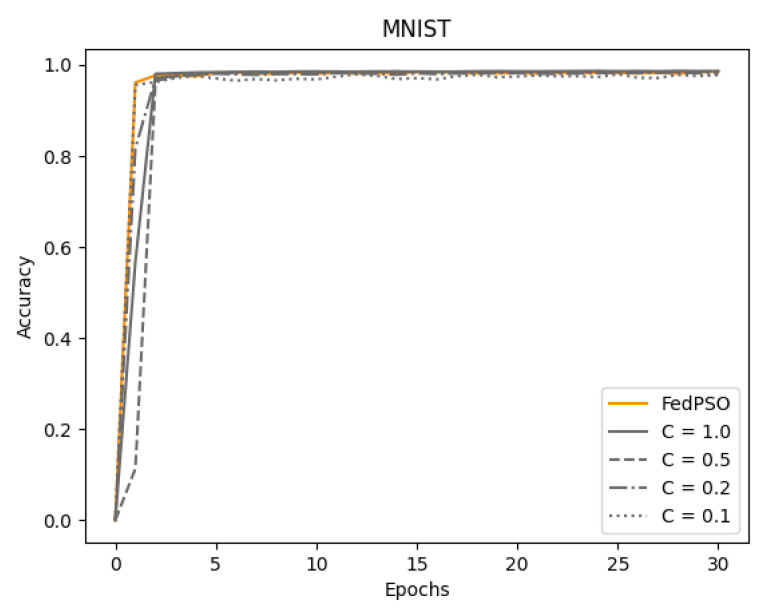
Comparison of learning accuracy using MNIST.

The accuracy experimental results with the MNIST dataset are presented in Figure 6 and Table 4. The MNIST dataset results in good performance even in a model with a small number of layers. Therefore, even if the size of the model was not sufficient, such as MNIST, there was no significant difference between FedPSO and FedAvg. As shown in Figure 6 and Table 4, the difference in accuracy between the two algorithms is negligible, approximately 0.1%. However, in the case of FedPSO, convergence occurs in fewer epochs.

### 4.3. Experimental Result for Unstable Network Environment

**Figure 7:**
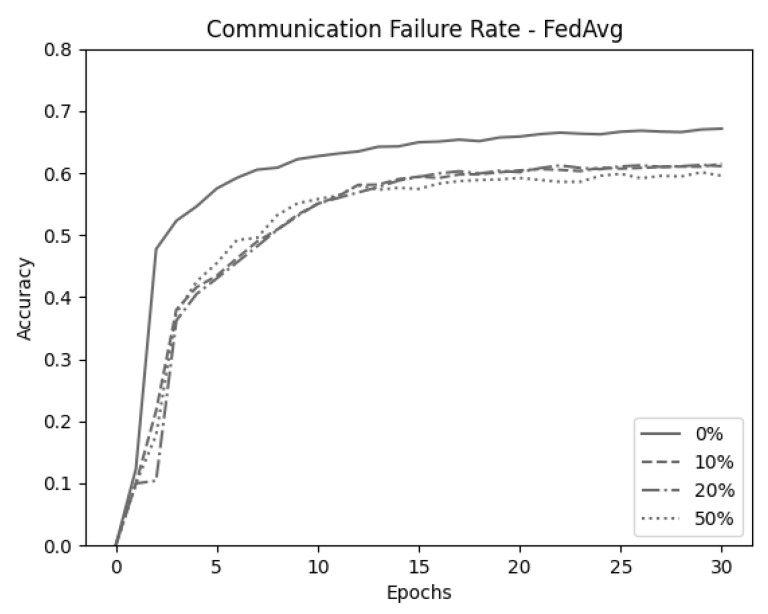
Comparison of FedAvg (*C* = 1.0) test accuracy in unstable network conditions.

**Figure 8:**
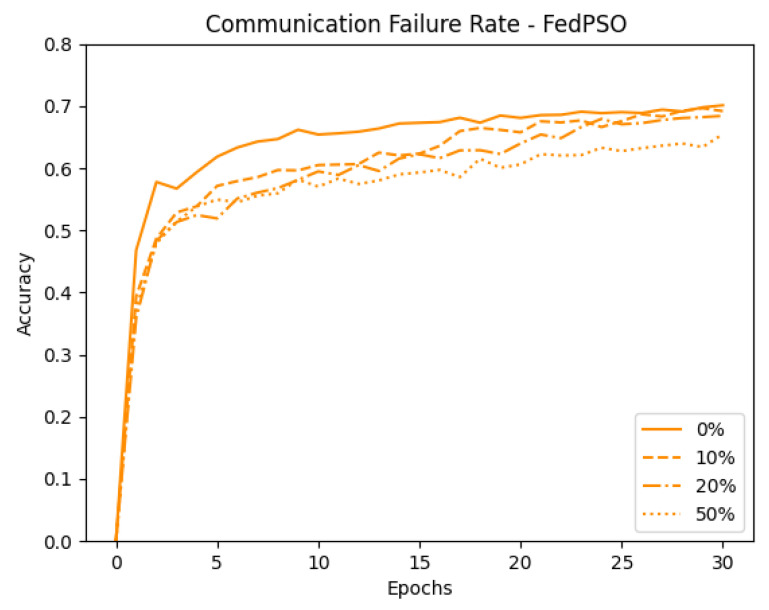
Comparison of FedPSO test accuracy in unstable network conditions.

Next, we emulated an unstable network environment. Data transmitted from client to server were dropped randomly in each communication round. To confirm the difference in accuracy between the two algorithms in this environment, data were dropped in the ranges of up to 0%, 10%, 20%, and 50%. Finally, for the validity of the experiment, all experiments were conducted through the average value after 10 experiments. Figure 7 shows the result of randomly dropping data for FedAvg when C=1.0. FedAvg shows an average decline in accuracy of 6.43% caused by the random data drops. Figure 8 shows the results for FedPSO, which experienced an average accuracy decrease of 2.43%. Detailed accuracy results are given in Table 5. In the experiment testing the model in an unstable network environment in which the data cannot be transmitted completely, FedPSO’s accuracy reduction better than FedAvg by 4%.

## 5. Conclusions

This study proposed a particle swarm optimization-based FedPSO algorithm to improve the network communication performance of federated learning and reduce the size of data sent from clients to servers. The proposed algorithm aggregates the model trained on the server by sharing the score value. The client with the best score provides the trained model to the server. The proposed algorithm was trained on the CIFAR-10 datasets through a two-layer CNN. On average, it produced an accuracy improvement of 9.47% over FedAvg and an accuracy improvement of 5.12% in experiments when communication costs were similar. When the same number of clients were used for training, the accuracy improved by 2.98%, even when the network communication cost was greatly reduced to the 55% level. The results showed that FedPSO can perform federated learning even in situations in which network communication is unstable and it is difficult to send large amounts of data to servers. In addition, when communication data are randomly dropped, FedPSO is on average 4% more robust than FedAvg. However, in a model that does not require a deep layer, such as MNIST, there was no significant difference between the two algorithms.

In the future, we plan to apply diverse forms of PSO to improve network communication performance. For example, we will study how to reduce the probability of falling into local minima by using dynamic multi-swarm PSO and allow client P2P communication using P2P-PSO. For further network communication efficiency with frequent client drops and limited network bandwidth, we plan to apply diverse network protocols such as the gossip protocol [14]. Moreover, as described above, when the ANN layer increases, the size of the model increases proportionally. Therefore, we plan to experimentally verify the results that can be displayed for each layer size in a model using deeper layers in the future.

## Figures and Tables

**Table 1:** Parameters settings for the CNN.

Layer	Shape
Conv2D	5 × 5 × 32
Conv2D	32
Conv2D	5 × 5 × 64
Conv2D	64
Dense	1024 × 512
Dense	512
Dense	512 × 10
Dense	10

**Table 2:** The constant of our proposed model.

	FedAvg	FedPSO
Client	10	10
C	0.1, 0.2, 0.5, 1.0	-
Epoch	30	30
Client-epoch	5	5
Batch	10	10
α	-	0.3
c1	-	0.7
c2	-	1.4

**Table 3:** Test Accuracy.

	Test Accuracy.
FedPSO	**70.12%**
FedAvg, *C* = 1.0	67.14%
*C* = 0.5	65.00%
*C* = 0.2	59.07%
*C* = 0.1	51.39%

**Table 4:** Test Accuracy.

	10 Epochs	20 Epochs	30 Epochs
FedPSO	98.10%	98.23%	98.55%
FedAvg, *C* = 1.0	98.58%	98.61%	98.65%
*C* = 0.5	98.31%	98.46%	98.61%
*C* = 0.2	97.95%	98.22%	98.07%
*C* = 0.1	96.83%	97.47%	97.80%

**Table 5:** Difference in accuracy according to the probability of communication failure.

	Failure Rate 0%	10%	20%	50%
FedPSO	**70.12%**	**69.18%**	**68.41%**	**65.47%**
FedAvg, *C* = 1.0	67.14%	61.48%	61.09%	59.55%

## Data Availability

FedPSO GitHub.
